# Lectures on Surgery, Delivered at the Philadelphia Medical Institute

**Published:** 1838-05-09

**Authors:** Thomas Harris

**Affiliations:** Surgeon to the Pennsylvania Hospital, &c.


### LECTURES ON SURGERY, *delivered at the Philadelphia Medical Institute, by* THOMAS HARRIS, M. D., *Surgeon to the Pennsylvania Hospital, &c.*

### ULCERS.

IN common language an ulcer and sore are used as synonymous. The term ulcer is derived from the Greek word Ελκος, signifying to draw, because it was thought that peccant and unhealthy humours of the body were eliminated through it. Hence the old practice of dressing sores with such stimulating salves, as promote purulent discharges. This doctrine is now happily repudiated. I have already told you that suppuration is a destructive process, depending on derangement in the action of the capillaries, which secrete, in a healthy state, both the solids and interstitial fluids of the body. Ulceration is a mere compound action, consisting of the formation of little organic or fleshy eminences, called granulations, connected with the secretion of pus. The class of capillaries that secrete the solids of the body are now engaged in building granulations, while those capillaries, which in their natural state secrete the interstitial fluids during the process of ulceration, secrete pus. Burns and other surgical pathologists are of opinion, that when there are no granulations, perfect or imperfect, healthy or the contrary, there can be no ulceration. Ulceration is a restorative process, in which healthy flesh, or granulations, are secreted. Where there are no granulations, it is a "mere suppurating surface." Although I concur in opinion with these gentlemen, still I will for the sake of custom and convenience continue the old nomenclature.

Ulcers are divided into healthy or simple, indolent, irritable, and phagedenic. The simple healthy ulcer is commonly the result of an abscess, and is met with in a sound constitution. Its characters are florid and pointed granulations, having an even surface, but slightly elevated above the surrounding skin, and covered by a matter of the colour and consistence of cream. By granulations, I mean those little eminences springing from the cellular tissue, by which the surface of an ulcer is covered. They present a reticulated structure when examined through a microscope. Their bases are broad, and they contract as they approach the surface, to about one-third of their original diameter. When the constitution is sound, and the body in a healthy condition, they spring up very rapidly. They evince a great disposition to unite one with another, and from this natural process we derive useful hints in their treatment.

When the solution of continuity is entirely filled up by red, and even granulations, secreting a yellowish pus, the process of cicatrization commences. A white, shining transparent film covers the surface of the sore. The cutis vera is first formed, the cuticle next, and the rete mucosum last of all. Thus we can account for the difference of color in a cicatrix which often exists so long. This is peculiarly the case with the negro. Sometimes indeed the rete mucosum is never regenerated.

Of all the remedies which have been proposed to cure ulcers not one deserves the name. Ulceration is a natural, restorative process, instituted to repair some injury. All that the surgeon can do is to assist the healthy action which is going on. A poultice at first will be found a very soothing and comfortable application. When the granulations have risen near the surface, the poultice should be removed; it relaxes and weakens the parts, and now does harm. Simple cerate, adhesive plaster, or dry lint may be substituted. An oval piece of dry lint may be applied to the centre of the sore. Sometimes the granulations become too luxuriant, and are said to be *fungous*, or are termed in common language *proud-flesh.* In such cases they may be compressed by the means just mentioned. When languid we must apply some stimulating application. Numerous ointments have been proposed to effect this. These greasy substances are very apt to irritate the sore, on account of their becoming rancid from the heat of the parts. I therefore, commonly prefer using the same articles in the form of washes. These may be applied on a piece of lint to the surface of the ulcer, and the whole covered by oiled silk to prevent evaporation. The black and yellow washes, solutions of the nitrate of silver, and sulphate of copper may be used for this purpose. Attention must be paid to the diet of the patient, as well as to his general health, and all stimulating drink and food must be forbidden. The part must also be kept at rest. I may mention that within a few years, in the London Hospitals, cold water has been found a very pleasant and excellent application to ulcers.

Sometimes, from bad treatment, or from impaired constitutional powers, the ulcer assumes an indolent character, evincing an indisposition to heal. There are no granulations; the surface is flat and shining, glassy and semi-transparent. The edges are smooth, rounded, elevated, and protuberant, making the chasm in the flesh appear much deeper than in reality it is; for it, in fact, is but little below the level of the skin. Indolent ulcers occur generally in parts remote from the centre of the circulation, as the leg; and are most frequently met with in intemperate habits. Local means will effect but little, unless the constitution is attended to. You must first regulate this. A good pill to improve the secretions, and conduce towards this end, is the following:—

℞. Extract. colocynth. comp. gr. xxiv.
Pil. hydrarg.
Pulv. rhei, *aa* gr. xij.
Ft. mass. et div. in pil. no. xii.
Sig. Two or three at night.

Where gastric derangement exists, as we often find in persons of luxurious habits of life, I have found no mixture so excellent as the following. Indeed, in several forms of dyspepsia, especially in those connected with irritation of that viscus, I look upon it as invaluable:

℞. Extract. taraxici, ℥j.

Potassæ tart. ʒvj.

Sodæ bi-carb. ʒj.

Tinct. rhei, f.ʒvi.

Aq. bullient. O j.

M. ft. infus.

Sig. A half a wine-glass full three times a day.

Place your patient in bed, elevate his limb, and apply a poultice. You must then employ stimulating applications. Of these there are a great number, and you will find it advantageous to be acquainted with, and employ perhaps all. It is very necessary to change frequently your applications in the treatment of all kinds of ulcers. An ulcer will do very well for some days under one application, which will then lose its effects, and you must resort to another, and another, until you succeed in accomplishing a cure. The fermenting poultice, made by mixing Indian meal and porter, and putting them before the fire to ferment; poultices containing the chloride of lime or soda; the black and yellow wash, and the solutions of the sulphate of copper or zinc, with a host of others, may be mentioned. A solution of nitric acid,—fifty drops to the pint of water,—is highly recommended by Sir Astley Cooper, in this form of ulcer. An excellent application to an indolent ulcer, and one which I frequently employ, consists of equal parts of bees-wax and Venice turpentine, melted together, and poured, when cooling, into the ulcer, and confined there by strips of adhesive plaster. So long, however, as the edges remain in the callous and undermined condition before mentioned, it is impossible to cure the ulcer. They must be removed by the knife or caustic. The method usually employed, is to apply over the ulcer a piece of adhesive plaster, cut to fit the sore, and then to burn off the edges with the caustic potash. A plan of treatment for indolent ulcers was proposed some years ago by Baynton. It consisted of the application of adhesive straps, encircling three-fourths of the leg, with holes cut in them to permit the passage of the matter. This plan sometimes succeeds very well, but I have seen it prove very injurious. Baynton says, that by adopting this method the necessity of confinement to bed is obviated, he allowing the patient to walk about. I never saw yet a case of ulcer where motion did not do harm. One variety of the indolent ulcer, and a very common form of the disease, is connected with an enlarged or varicose condition of the veins. This is the result of phlebitis, or inflammation of the veins, for in every case we find them preternaturally thickened; they are four times as thick, and often twice as long as natural. The veins are very tortuous, and return on themselves. The valves do not act, and the column of blood has nothing to sustain it. Ambrose Paré, and the old surgeons, were in the habit of removing the enlarged venous cluster, by the actual cautery. This was, of course, a very cruel and unnecessary procedure. Another practice is to cut down and apply a ligature to the vessel. This is a very dangerous operation, fatal phlebitis often following it. Sir Astley states, that he would, in his own case, rather have a ligature applied to his femoral artery, than have his saphena vein tied. Sir Benjamin Brodie proposes to divide the vein; for this purpose he introduces a narrow, slightly curved bladed bistoury with its cutting edge on the convex side, between the integument and vein, and turning the back to the former, cuts through the vessel. Reunion, however, is found to follow this operation, and the varicose condition to return. In the early stage, leeching along the course of the vein, aided by compression, is often sufficient to effect a cure. If the disease has existed for any time, an operation becomes necessary. The one I have been in the habit of performing, is that proposed originally by Dr. Hartshorne of this city. You cut down upon the vein, dissect out about two inches, and remove it, you then apply a compress above and below the wound, and confine the whole limb by a bandage. The first dressings are to be suffered to remain four or five days. I have now performed this operation fifteen times, and in but one instance did any bad effects follow. That patient had an attack of phlebitis, from which he recovered. The French surgeons have lately proposed a new operation for this affection; it consists in passing a needle through the vein and confining it there for several days by means of a ligature. I have tried it lately in a case of varicocele, and with success. On the same principle, Fricke, of Hamburg, passes a ligature through the vein, and permits it to remain in a sufficient length of time to excite the requisite inflammation for the obliteration of the vessel. Liston, and other English surgeons, apply caustic to that portion of the vein which is healthy, until inflammation occurs and its cavity is destroyed.

The next description of ulcers to which I shall direct your attention is the irritable ulcer. This may be recognised by the great pain it occasions, the jagged, irregular edges; the florid, unequal granulations, and the bloody, fœtid and ichorous discharge. The constitutional symptoms too are often very severe and distressing. Pressure on the part occasions intense suffering; the weight even of a poultice will sometimes produce a great deal of pain. Various local applications have been recommended; among these, fomentations with poppy heads will be found very soothing and serviceable. The mucilages of flax seed, slippery elm, and sassafras, you will also find very advantageous when inflammation exists; indeed, when this occurs to any extent, you must resort to leeching; your leeches must be applied around the ulcer, to the sound uninflamed skin. Sir Astley Cooper's anodyne lotion you will find, at times, a valuable application. We use it in the Pennsylvania Hospital with a great deal of success. It is composed of

℞. Extract. opii, ʒss.

Pulv. acac. ʒiss.

Aq. calcis, ℥ivss.

M. ft. sol.

But the great secret in the treatment of every description of ulcer, as I have before told you, is to change constantly your applications; when you find one losing its effects, try another, and so on. In the treatment of indolent ulcers the greatest benefit is derived from the internal use of opium and calomel. Some surgeons are of opinion that the anodyne alone will answer, but my experience is in favour of the addition of the mercurial. I give one grain of opium and two of calomel twice or thrice a day. From the employment of this remedy I have derived the greatest benefit. When the character of the ulcer is changed, and the granulations begin to spring up, the local application of opium must be stopped, as it tends to deaden the parts, and prevent the healing process.

The last variety of ulcers of which I shall speak to-day, is the sloughing or phagedenic ulcer. The phagedenic ulcer prevails often to a great extent in hospitals. In the Pennsylvania Hospital, last winter, there must have been at least twenty cases. It often proves very fatal, attacking patients of all descriptions, causing a fatal termination to the slightest wound. When it attacks a part, the granulations lose their florid hue and become flabby; the parts swell, and an ichorous discharge is poured out. It is commonly connected with erysipelas. Some surgeons consider it as contagious. The constitution is severely implicated; the pulse can scarcely be felt; the countenance becomes sunken; the eyes are glassy; a cold sweat covers the whole body, and the patient rapidly sinks. Various means have been proposed to arrest this formidable malady. In the Pennsylvania Hospital the purgative plan has been found the most effectual, combined with the usual local means for the arrest of gangrene. Blisters have been highly lauded as a means of arresting hospital gangrene, but I have known repeatedly the parts on which the blister has been applied to slough, and thus aggravate the patient's condition. Removal to another ward will very often speedily put a stop to this affection.

